# Investigation of SARS-CoV-2 infection and associated lesions in exotic and companion animals

**DOI:** 10.1177/03009858211067467

**Published:** 2022-01-18

**Authors:** David S. Rotstein, Sarah Peloquin, Kathleen Proia, Ellen Hart, Jeongha Lee, Kristin K. Vyhnal, Emi Sasaki, Gayathriy Balamayooran, Javier Asin, Teresa Southard, Laura Rothfeldt, Heather Venkat, Peter Mundschenk, Darby McDermott, Beate Crossley, Pamela Ferro, Gabriel Gomez, Eileen H. Henderson, Paul Narayan, Daniel B. Paulsen, Steven Rekant, Megan E. Schroeder, Rachel M. Tell, Mia Kim Torchetti, Francisco A. Uzal, Ann Carpenter, Ria Ghai

**Affiliations:** 1 US Food and Drug Administration, Center for Veterinary Medicine, Office of Surveillance and Compliance, Rockville, MD, USA; 2 US Food and Drug Administration, Center for Veterinary Medicine, Office of Research, Veterinary Laboratory Investigation and Response Network, Laurel, MD, USA; 3 Louisiana State University, Baton Rouge, LA, USA; 4 Texas A&M Veterinary Medical Diagnostic Laboratory, College Station, TX, USA; 5 University of California–Davis, San Bernardino, CA, USA; 6 Cornell University, Ithaca, NY, USA; 7 Arkansas Department of Health, Zoonotic Disease Section, Little Rock, AR, USA; 8 Center for Preparedness and Response, Centers for Disease Control and Prevention, Atlanta, GA, USA; 9 Arizona Department of Health Services, Phoenix, AZ, USA; 10 Arizona Department of Agriculture, Phoenix, AZ, USA; 11 New Jersey Department of Health, Communicable Disease Service, Trenton, NJ, USA; 12 USDA APHIS Veterinary Services, Riverdale, MD, USA; 13 USDA National Veterinary Services Laboratories, Ames, IA, USA; 14 Centers for Disease Control and Prevention, Atlanta, GA, USA

**Keywords:** bronchopneumonia, dogs, COVID-19, cats, respiratory, severe acute respiratory syndrome coronavirus 2, syncytial cell, viral, tiger, zoonosis

## Abstract

Documented natural infections with severe acute respiratory syndrome coronavirus 2 (SARS-CoV-2) in exotic and companion animals following human exposures are uncommon. Those documented in animals are typically mild and self-limiting, and infected animals have only infrequently died or been euthanized. Through a coordinated One Health initiative, necropsies were conducted on 5 animals from different premises that were exposed to humans with laboratory-confirmed SARS-CoV-2 infection. The combination of epidemiologic evidence of exposure and confirmatory real-time reverse transcriptase-polymerase chain reaction testing confirmed infection in 3 cats and a tiger. A dog was a suspect case based on epidemiologic evidence of exposure but tested negative for SARS-CoV-2. Four animals had respiratory clinical signs that developed 2 to 12 days after exposure. The dog had bronchointerstitial pneumonia and the tiger had bronchopneumonia; both had syncytial-like cells with no detection of SARS-CoV-2. Individual findings in the 3 cats included metastatic mammary carcinoma, congenital renal disease, and myocardial disease. Based on the necropsy findings and a standardized algorithm, SARS-CoV-2 infection was not considered the cause of death in any of the cases. Continued surveillance and necropsy examination of animals with fatal outcomes will further our understanding of natural SARS-CoV-2 infection in animals and the potential role of the virus in development of lesions.

Zoonotic transmission of severe acute respiratory syndrome coronavirus 2 (SARS-CoV-2) has been documented globally but is not considered to play a significant role in driving the COVID-19 (coronavirus disease 2019) pandemic. Sustained transmission in animal populations has been a concern due to the risks of mutations or variants emerging in animals, as well as the potential establishment of new animal reservoirs of infection. A growing body of research has investigated the zoonotic nature of SARS-CoV-2 transmission using a One Health approach.^7,8,16–18^ Globally, natural infections from infected animals or people with SARS-CoV-2 have been reported in dogs, cats, gorillas, exotic felids (lions, tigers, snow leopards, cougars), ferrets, otters, and mink.^1,12,16–23^ Most often, animals become infected with SARS-CoV-2 after exposure to a person with COVID-19. Companion animals are commonly infected by their owners, while exotic animals and farmed animals like mink are commonly infected by caretakers.^17,19^ Infected animals have presented with clinical signs including fever, coughing, sneezing, difficulty breathing, lethargy, oculonasal discharge, vomiting, and diarrhea.^6^

Early in the pandemic, the One Health Federal Interagency COVID-19 Coordination (OH-FICC) group was established to facilitate joint US government action and collaboration on One Health issues pertaining to SARS-CoV-2. The OH-FICC is composed of over 130 individuals from 22 US federal agencies and is chaired by the US Centers for Disease Control (CDC) One Health Office. Five OH-FICC subgroups were created: companion animals, animal diagnostics and testing, wildlife and zoo animals, livestock and production animals, and environmental health. Subgroups have varying responsibilities, including surveillance, reporting, and guidance development.^24^

In cases where animals were determined to be infected with SARS-CoV-2 and had fatal outcomes, necropsies were initially performed without standardized processes in place. Some institutions faced logistical and financial challenges. To address these challenges, the OH-FICC’s Animal Diagnostic and Testing Subgroup initiated necropsy coordination and assistance through the Food and Drug Administration’s (FDA) Veterinary Laboratory Investigation and Response Network (Vet-LIRN) and its 46 network laboratories in North America composed of veterinary medical colleges and state diagnostic laboratories. To address consistency of sampling and tracking, a necropsy sampling checklist was developed and posted on FDA’s public website for laboratory use.^9^

We detail findings from 3 cats (cases 1–3) and 1 dog (case 5) necropsied using these coordination efforts, as well as 1 tiger (case 4) that was necropsied by a Vet-LIRN laboratory separately from this coordination process. Four animals (cases 1–4) had positive real-time reverse transcriptase-polymerase chain reaction (rRT-PCR) test results. Case 5 tested negative with rRT-PCR and virus neutralization, but was included to document the pathological findings in an animal that met testing recommendations (exposure to SARS-CoV-2 and presence of clinical signs).

## Materials and Methods

Testing for cases involved initial testing at a private laboratory (cases 1, 2, 5) or directly at USDA NVSL (cases 3, 4), which served as the confirmatory laboratory (Supplemental Tables S1 and S2). Testing at the private laboratory was conducted after consultation with state health officials.^24^ The private laboratory’s rRT-PCR targeted the same nucleocapsid genes (2019-nCoV-N1 and 2019-nCoV-N2)^13^ as used in the Centers for Disease Control and Prevention (CDC) assays^5,14^; the private laboratory rRT-PCR test was validated through a comparison to 3 CDC assays.^5,14^ Based on the presumptive positive results, samples from cases 1 and 2 were confirmed at the US Department of Agriculture (USDA) National Veterinary Services Laboratories (NVSL) by the CDC 3-panel N-target rRT-PCR test (Supplemental Materials—Materials and Methods).^5,11,14^ Briefly, The MagMAX-96 Viral RNA Isolation Kit (ThermoFisher Scientific) was used to extract RNA. The rRT-PCR N-target assay was used on an Applied Biosystems 7500 Fast Real-Time PCR instrument as described in the Emergency Use Authorization from CDC.^5^ The rRT-PCR amplification was performed with 1 cycle at 50 °C for 15 minutes and at 54 °C for 10 minutes, followed by 40 cycles of 95 °C for 15 seconds and 55 °C for 1 minute on an Applied Biosystems 7500 Fast Real-Time PCR Instrument. Viral whole genome sequencing (WGS) was conducted on cases 2 to 4 from nucleic acid from rRT-PCR-positive specimens extracted and sequenced using the Oxford Nanopore Technologies 78 MinION and Illumina MiSeq; consensus sequences were generated with 79 Minimap 2.17 and Samtools 1.9 (Supplemental Materials—Methods).^11^ Virus neutralization was performed on serum from cases 4 and 5; virus neutralizing titers of ≥1:32 are considered confirmatory.^25^ Briefly, 2-fold serially diluted sera for a final dilution of 1:8 to 1:512 were incubated with TCID50/mL SARS-CoV-2 for 60 minutes. Vero cells were added to the virus-serum mixtures, and titers were determined at 3 days post infection. Titer recording was determined by the reciprocal of the highest serum dilution that provided 100% neutralization of the reference virus, as determined by visualization of cytopathic effect (Supplemental Materials—Methods). Cases were considered positive based on the case definition established by the USDA^25^ that included suspect, presumptive positive, and confirmed positive cases. Cases 1 to 4 were confirmed positive based on a combination of the following criteria: epidemiologic link with a confirmed human COVID-19 patient, positive SARS-CoV-2 rRT-PCR assay, and sequence confirmation. Case 5 was suspect based on the criteria of an epidemiologic link with a confirmed human COVID-19 patient.

Full necropsies were performed on all animals, and representative sections were fixed in 10% formalin, processed routinely, sectioned at 5 to 7 µm, and stained with hematoxylin and eosin. For case 5, lung sections were also stained with Masson’s trichrome and phosphotungstic acid-hematoxylin (PTAH). SARS-CoV-2 rRT-PCR was conducted on formalin-fixed paraffin-embedded (FFPE) sections of lung from cases 4 and 5 as described by Bhatnagar et al.^2^ Immunohistochemistry (IHC) for SARS-CoV-2 was conducted on FFPE lung sections from case 4. IHC involved a rabbit polyclonal antibody against SARS-CoV nucleocapsid (Novus Biological, catalog number NB100-56576) at a 1:100 dilution and a Mach 4 Universal AP Polymer Kit and Permanent Red Chromogen. Slide pretreatment was done with a heat-induced epitope retrieval with a citrate buffer. Cross-reactivity was assessed with influenza, parainfluenza, and MERS-CoV, and cross-reactivity was not observed.^15^

## Results

Case signalment, diagnostic, and necropsy findings are provided in Table 1, and additional information is provided in Supplemental Tables S1 and S2. The cases originated from Arkansas (cats, cases 1 and 2), New Jersey (cat, case 3), Texas (tiger, case 4), and Arizona (dog, case 5). All cases were epidemiologically linked to a human case that tested positive for COVID-19, and clinical signs in the animals developed within 2 to 12 days (mean, 6 days) after the person was sampled.

**Table 1.** Clinical, diagnostic, and pathologic findings in animals exposed to humans with laboratory-confirmed SARS-CoV-2 infection.

Case	Species	Age (Years)	Sex	rRT-PCR-Positive Tissues	Genotype	Clinical Signs	Necropsy Findings
1	Cat	13	F	NS, TS, PS	ND^a^	SD	MMC
2	Cat	0.8	MN	NS (AM)	Clade 20G (B.1.2)	ARF, OND	RF, RO
3	Cat	18	FS	NS	Clade 20C (B.1.526)	DYS, HTH, OND	CIN, LVH
4	Tiger	20	F	FEC (AM)	Clade 20A (B.1.234)	COU, NDI, PNEU	BBP, SC
5	Dog	10	MN	NEG	—	COU, DIA, OND, SNE	BIP, SC, DCM, BAS

Abbreviations: AM, antemortem; ARF, acute renal failure; BAS, brachycephalic airway syndrome; BBP, bacterial bronchopneumonia; BIP, bronchointerstitial pneumonia; CIN, chronic interstitial nephritis; COU, cough; DCM, dilated cardiomyopathy; DIA, diarrhea; DYS, dyspnea; FEC, fecal; HTH, hypothermia; LVH, left ventricular hypertrophy; MMC, metastatic mammary carcinoma; ND, not done; NDI, nasal discharge; NEG, negative; NS, nasal swab; OND, oculonasal discharge; PNEU, pneumonia; PS, pharyngeal swab; RO, renal oxalosis; RF, renal fibrosis; rRT-PCR, real-time reverse transcriptase-polymerase chain reaction; SC, syncytial-like cells; SD, sudden death; SNE, sneezing; TS, tracheal swab.

^a^ Sequencing of the nucleic acid sample was done for confirmatory testing. Whole genome sequencing could not be done due to sample limitations.

Case 1 was a 13-year-old intact female cat in an animal shelter that had come from a household with 2 dogs. The cat was found dead with no premonitory clinical signs. The cat was frozen, and several collected specimens tested positive by SARS-CoV-2 rRT-PCR (Supplemental Tables S1 and S2). Gross findings included mammary masses and pulmonary nodules. The cat had metastatic mammary carcinoma that spread to the lung and lymph nodes, thyroid gland atrophy, and hepatic stellate cell hyperplasia. Due to specimen limitations, WGS was not performed. Rectal and oropharyngeal swabs from 2 asymptomatic dogs living in the same household were negative by rRT-PCR. SARS-CoV-2 virus neutralization titers for these dogs were 1:32 and 1:128 (positive).

Case 2 was a 9-month-old male neutered cat. The cat was attacked by a dog and presented with oculonasal discharge and acute renal failure. A nasal swab tested positive for SARS-CoV-2 by rRT-PCR, and WGS identified genotype B.1.2. The cat was reevaluated 2 months later and euthanized due to progressive renal disease. At necropsy, tracheal, oropharyngeal, and rectal swabs were negative by rRT-PCR. There was bilateral hydronephrosis, and histopathologic findings included chronic lymphoplasmacytic interstitial nephritis, interstitial fibrosis, tubular degeneration, intratubular oxalate crystals, and multi-organ vascular mineralization.

Case 3 was an 18-year-old female spayed cat with a history of medically managed hyperthyroidism and atopic skin disease. The cat presented with hypothermia, oculonasal discharge, and dyspnea. Radiographically, there was a diffuse interstitial lung pattern. The cat was euthanized due to a poor prognosis. A nasal swab collected after death tested positive for SARS-CoV-2 by rRT-PCR, and WGS identified variant B.1.526. Grossly, there was pulmonary edema and concentric hypertrophy of the left ventricle of the heart. Histopathologic findings included pulmonary edema with type II pneumocyte hyperplasia, lymphoplasmacytic interstitial nephritis, and pancreatic nodular hyperplasia. No histopathologic changes were observed in the heart.

**Figure 1.**
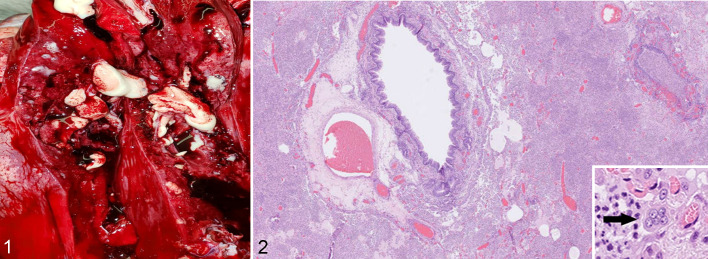
Suppurative pneumonia, lung, tiger, case 4. On cut section, purulent exudate is present within bronchial lumina. The lung is consolidated and congested. **Figure 2.** Suppurative streptococcal bronchopneumonia, lung, tiger, case 4. Alveoli are filled with inflammatory cells including neutrophils, macrophages, and few syncytial-like cells (inset). Hematoxylin-eosin.

Case 4 was a 20-year-old female tiger from a zoological park. The tiger developed a progressively worsening cough, nasal discharge, and pneumonia, and was euthanized. An antemortem fecal swab was positive for SARS-CoV-2 by rRT-PCR, and WGS identified genotype B.1.234. At necropsy, the lung was consolidated and purulent exudate was present in bronchioles (Fig. 1). Histopathologic findings included suppurative bacterial bronchopneumonia. Bronchiolar and alveolar spaces contained viable and degenerate neutrophils, macrophages, necrotic cellular debris, and bacterial cocci. In addition, within alveolar spaces, there were few syncytial-like cells (Fig. 2) as well as type II pneumocyte hyperplasia and fibroblast proliferation. A lung swab culture yielded growth of *Streptococcus equi subsp. zooepidemicus*. SARS-CoV-2 rRT-PCR was negative, SARS-CoV-2 IHC was negative, *Streptococcus* spp. IHC was performed to provide additional supportive evidence of the streptococcal pneumonia. The *Streptococcus* spp. IHC utilized a *Streptococcus pneumoniae* mouse monoclonal antibody (ThermoFisher, catalog number MA1-8347) with prior laboratory observed cross-reactivity (Supplemental Materials—Methods)^15^; *Streptococcus* spp. IHC was positive. Screening for viral and non-streptococcal bacterial agents was negative (Supplemental Table S1).

Case 5 was a 10-year-old male neutered pug dog from a household of 3 dogs that presented with an acute onset of coughing, sneezing, oculonasal discharge, and diarrhea. Respiratory distress worsened and the dog was euthanized. Nasal and lung swabs tested negative by SARS-CoV-2 rRT-PCR. SARS-CoV-2 virus neutralization was negative. Two other dogs in the household were SARS-CoV-2 negative by rRT-PCR nasal swabs but had virus neutralizing titers of 1:16 (negative) and 1:128 (positive). At necropsy, airway changes consistent with brachycephalic airway syndrome were evident including stenotic nares, elongated soft palate, everted laryngeal saccules, and decreased tracheal diameter. There was biventricular cardiac dilation. Histopathologic findings included lymphoplasmacytic bronchointerstitial pneumonia with septal thickening, fibrin lining alveolar septa, type II pneumocyte hyperplasia, and syncytial-like cells (Suppl. Figs. S1–S3) as well as cardiomyocyte atrophy and myocardial fatty infiltration. In the lung, PTAH-positive polymerized fibrin was present in pulmonary vessels and few alveolar spaces; Masson’s trichrome highlighted mild alveolar septal fibrosis. Viral pathogen testing and SARS-CoV-2 rRT-PCR from FFPE lung and kidney were negative. Based on the results of the rRT-PCR on FFPE tissues, SARS-CoV-2 immunohistochemistry was not conducted.

## Discussion

Other than exposure to a SARS-CoV-2-infected human, cases had limited commonalities except for respiratory clinical signs (cases 2–5). A standardized algorithm for animal assessment was applied to all SARS-CoV-2-positive cases in this study to assess clinical signs, presence of comorbidities, presence of SARS-CoV-2 in relevant specimens, and attributable histopathological changes.^4^ Based on this algorithm, SARS-CoV-2 infection was not considered as the primary reason for euthanasia or death in any of the cases, similar to the findings of the study in which the algorithm was developed.^4^ Comorbidities in the current SARS-CoV-2-confirmed cases included metastatic mammary carcinoma, myocardial disease, renal disease, and bacterial bronchopneumonia.

From prior reports, symptomatic SARS-CoV-2 infected dogs and cats have had transitory respiratory clinical signs as observed in cases 2 to 4.^6,10,20^ Self-limiting infection was supported by case 2, in which testing for SARS-CoV-2 was negative 2 months after the initial confirmation and there were no gross or histopathologic lesions attributed to viral infection. The timing of viral clearance is not known because no subsequent antemortem testing was done after initial diagnosis. In experimental studies in domestic cats at day 21 post-exposure, viral nucleic acid could not be detected and tracheobronchoadenitis was the sole histopathologic finding in the respiratory system.^10^ Tracheobronchoadenitis was not observed in any cases in the current study.

Syncytial-like cells and type II pneumocyte hyperplasia was present in cases 4 (rRT-PCR positive, feces) and 5 (rRT-PCR negative, nasal and lung). SARS-CoV-2 was not detected in the lung of either animal by rRT-PCR and/or IHC (case 4). In a human study, IHC using FFPE tissue had high specificity but less sensitivity than rRT-PCR.^20^ Lung lesions in case 4 were similar to reports from mink, a cat, and 2 dogs.^12,19,22^ Persistence of syncytial cells in the lung has been reported in humans with a biphasic course with initial viral replication and rRT-PCR positivity followed by hyperinflammation without detectable virus.^3^ While this may be the case with the dog (case 5) that was negative with multiple tests, other considerations for the pulmonary syncytia include chronic aspiration as a result of brachycephalic airway syndrome, existing heart disease, or other nonidentified infectious causes. Thus, bronchointerstitial pneumonia or bronchopneumonia with syncytial-like cells and hyaline membranes may not be sufficient as histopathologic evidence of SARS-CoV-2 infection. Exposure of animals did occur in the household of case 5 given that 1 of 2 asymptomatic dogs had a serum virus neutralizing titer of >1:32.

Three SARS-CoV-2 genotypes were identified from cases 2 to 4 (Table 1). WGS was not done on samples from the epidemiologically linked human cases to provide a molecular link to the human exposure.

The 5 cases presented are linked by exposures to people with positive test results for SARS-CoV-2 and not by gross, histopathological, temporal, spatial, or genotypic findings. However, the limited number of cases precludes population-based extrapolation. While there are no common pathologic findings, the information gathered on these cases highlights the importance of necropsy and ancillary diagnostics in investigating fatal outcomes to provide insight into the possible role of SARS-CoV-2 infection. The coordinated One Health approach to necropsy and specimen tracking provides a model for future epidemic or pandemic responses to support animal health investigations.

## Supplemental Material

Supplemental Material, sj-pdf-1-vet-10.1177_03009858211067467 - Investigation of SARS-CoV-2 infection and associated lesions in exotic and companion animals Click here for additional data file. Supplemental Material, sj-pdf-1-vet-10.1177_03009858211067467 for Investigation of SARS-CoV-2 infection and associated lesions in exotic and companion animals by David S. Rotstein, Sarah Peloquin, Kathleen Proia, Ellen Hart, Jeongha Lee, Kristin K. Vyhnal, Emi Sasaki, Gayathriy Balamayooran, Javier Asin, Teresa Southard, Laura Rothfeldt, Heather Venkat, Peter Mundschenk, Darby McDermott, Beate Crossley, Pamela Ferro, Gabriel Gomez, Eileen H. Henderson, Paul Narayan, Daniel B. Paulsen, Steven Rekant, Megan E. Schroeder, Rachel M. Tell, Mia Kim Torchetti, Francisco A. Uzal, Ann Carpenter and Ria Ghai in Veterinary Pathology
