# Supplementary material for: Investigation of SARS-CoV-2 infection and associated lesions in exotic and companion animals
Source: Vet Pathol. 2022 Jan 18;59(4):707–11. doi: 10.1177/03009858211067467 (PMC9207983; doi:10.1177/03009858211067467)
Supplement: Supplemental Material, sj-pdf-1-vet-10.1177_03009858211067467 - Investigation of SARS-CoV-2 infection and associated lesions in exotic and companion animals [file sj-pdf-1-vet-10.1177_03009858211067467.pdf]

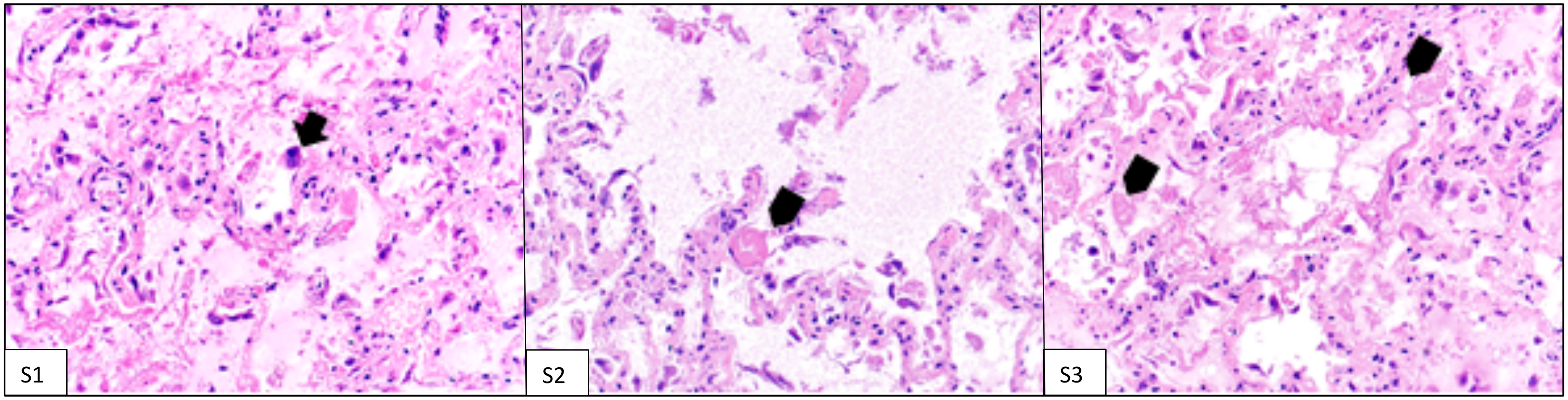

Supplemental Figure S1. Fibrinous pneumonia, lung, dog, case 5. Alveoli contain few- syncytial-like cells (arrow). HE.

Supplemental Figure S2. Vascular thrombosis, lung, dog, case 5. Within venules, there are occasional occlusive fibrin thrombi (arrow). HE.

Supplemental Figure S3. Fibrinous pneumonia, lung, dog, case 5. Fibrin is present within alveoli (arrow). HE.

Supplemental Table S1. Signalment, clinical signs, SARS-CoV-2 diagnostics testing, and necropsy findings for the SARS-CoV-2 positive PCR-confirmed Cases 1-4 and negative Case 5 that had exposure to COVID-19-confirmed people.

Case	Species	Age (years)	Sex	State	rRT-PCR	FFPE Lung -rRT-PCR or IHC	Genotype	Ancillary Diagnostics	Clinical Sign/ Days onset post human exposure	Gross and Histopathologic Findings
	Cat	13	F	AR	Nasal swab + (PM)<br>Tracheal swab + (PM)<br>Pharyngeal Swab + (PM)<br>Lung Swab – (PM)<br>Rectal swab – (PM)	ND	ND*	ND	Sudden death; no clinical signs (12d)	Metastatic mammary carcinoma,<br>Hepatic stellate cell hyperplasia,
2	Cat	0.8	MN	AR	Nasal swab + (AM)<br>Nasal swab – (PM)<br>Tracheal swab – (PM)<br>Pharyngeal swab – (PM)<br>Fecal swab – (PM)<br>Lung swab – (PM)	ND	Clade 20G (B.1.2)	neutrophilia-15K,<br>BUN >130 mg/dL,<br>creatinine 13.6 mg/dL,<br>Phosphorus >16.1	OND, ARF (7d)	Renal oxalosis<br>Renal fibrosis

Supplemental Table S1 (continued).

Case	Species	Age (years)	Sex	State	rRT-PCR	FFPE Lung -rRT-PCR or IHC	Genotype	Ancillary Diagnostics	Clinical Sign/ Days onset post human exposure	Gross and Histopathologic Findings
3	Cat	18	FS	NJ	Nasal swab + (PM)	ND	Clade 20C (B.1.526)	ND	HTH, DYS, OND (2d)	Left ventricular hypertrophy(gross observation), Pulmonary edema with type II pneumocyte hyperplasia Chronic interstitial nephritis
4	Tiger	20	F	TX	Nasal swab - (PM) Feces + (AM;)	NEG (IHC)	Clade 20A (B.1.234)	Bacterial culture, postmortem lung swab, *Streptococcus equi subsp zooepidemicus;* *Streptococcus* IHC lung +; SARS-CoV-2 IHC -; SARS-CoV-2 VN – 1:128 *Mycoplasma* spp. – Influenza A – *Chlamydia* spp. – *Bordetella bronchiseptica* – Feline herpesvirus – Feline calicivirus -	COU, NDI, PNEU (3d)	Streptococcal bronchopneumonia with syncytial-like cells and type II pneumocyte hyperplasia

Supplemental Table S1 (continued).

Case	Species	Age (years)	Sex	State	rRT-PCR	FFPE Lung -rRT-PCR or IHC	Genotype	Ancillary Diagnostics	Clinical Sign/ Days onset post human exposure	Gross and Histopathologic Findings
5	Dog	10	MN	AZ	Nasal swab – (PM)<br>Lung swab – (PM)	NEG (FFPE rRT-PCR)		CDV IHC –<br>PI3 FA –<br>Influenza PCR-<br>Bacterial culture, lung: *Enterococcus faecium*	COU, OND, SNE, DIA (5-6d)	Bronchointerstitial pneumonia with syncytial-like cells and type II pneumocyte hyperplasia<br>Cardiac biventricular dilation and myocardial fatty infiltration<br>Brachycephalic airway syndrome

Abbreviations: AM = antemortem, ARF = acute renal failure, CDV = Canine distemper virus, COU = cough, DIA = diarrhea, DYS = dyspnea, FFPE = formalin fixed paraffin embedded, HTH = hypothermia, ND = not done, NDI = nasal discharge, NEG = negative, OND = oculonasal discharge, PI 3 = Parainfluenza virus 3, PNEU = pneumonia, PM = post mortem, rRT-PCR = real-time reverse transcriptase polymerase chain reaction, SD = sudden death, SNE = sneezing, “+” = positive, “-“ = negative.

*Sequencing of the nucleic acid sample was done for confirmatory testing. Whole genome sequencing could not be completed due to sample limitations.

Supplemental Table S2. Results of rRT-PCR and virus neutralization. Preliminary test results were confirmed at USDA NVSL.

Case	rRT-PCR Result	rRT-PCR Method - Reference Number	Virus Neutralization Result (reference number)	Virus Neutralization Method (Reference Number)
1	Pos	13 (prelminary testing), 5,11 (confirmation)	Case 1 -NT; dogs in household -1:32 and 1:128	11
2	Pos	13 (preliminary testing),5,11 (confirmation),	ND	
3	Pos	5,11	ND	
4	Pos	5,11	1:128	11
5	Neg	13 (preliminary testing), 5, 11 (additional testing)	Case - Neg; dogs in household- 1:16 and 1:128	11

Abbreviations: ND = Not done, NEG = negative, NT = Not tested,

Supplemental Materials—Methods

Real-time Reverse Transcriptase Polymerase Chain Reaction (rRT-PCR)

The method described below if for sample testing at USDA NVSL from Goryoka et al. (2021).[11] For the private laboratory, we refer to the information on the private laboratories home page.[13]

RNA extraction and real-time reverse-transcription polymerase chain reaction (rRT-PCR) testing of animal specimens occurred at USDA NVSL.

RNA was extracted from 50 µL of sample using the MagMAX-96 Viral RNA Isolation Kit (Thermo Fisher Scientific) on a 24-well King Fisher extraction platform and eluted in a volume of 90 µL according to manufacturer's instructions. A modified CDC one-step rRT-PCR N-target assay [N1 and N2 targets] was used on an Applied Biosystems 7500 Fast Real-Time PCR Instrument according to Emergency Use Authorization instructions for use.. The rRT-PCR amplification was performed with 1 cycle at 50C for 15 mins and 54 C for 10 mins, followed by 40 cycles of 95C for 15 secs and 55 C for 1 min on an Applied Biosystems 7500 Fast Real-Time PCR Instrument.

Genome Sequencing

At CDC, nucleic acid from rRT-PCR positive specimens was extracted and sequenced using the Oxford Nanopore Technologies MinION and Illumina MiSeq following previously published protocols and consensus sequences were generated with Minimap 2.17 and Samtools 1.9. Missing gaps after MinION and MiSeq sequencing were filled by individual rRT-PCR followed by Sanger sequencing. Consensus complete genome sequences were generated using Sequencher 5.4.6.

Serum Virus Neutralization

For VN, 25µL of two-fold serially diluted sera (for final dilutions of 1:8 to 1:512) were pre-incubated with 25µL of TCID50/ml of SARS-CoV-2 (2019-nCoV/USA-WA1/2020) in MEM-E containing 200UI/mL penicillin, 200µg/mL 66 streptomycin, 75µg/ml gentamicin sulfate and 6µg/mL Amphotericin B for 60 min at 37°C with 5% CO2. Serum sample were tested in duplicate. One hour post-infection, 150µl of Vero cells were added to the virus-serum mixtures. The neutralization titers were determined at three days post infection. Titer recording was determined by the reciprocal of the highest serum dilution that provided 100% neutralization of the reference virus, as determined by visualization of cytopathic effect. The specificity of the VN assay was assessed in-house by testing sera with antibodies to transmissible gastroenteritis, porcine epidemic diarrhea virus, porcine hemagglutinating encephalomyelitis virus, bovine coronavirus, and Aleutian disease.

SARS-CoV-2 Immunohistochemistry

e conducted an IHC assay for SARS-CoV-2 using a rabbit polyclonal antibody raised against SARS-CoV nucleocapsid (Novus Biologicals, https://www.novusbio.com) (*19*) at 1:100 dilution and a Mach 4 Universal AP Polymer Kit (Biocare Medical, https://biocare.net) with Permanent Red Chromogen (Cell Marque/Millipore Sigma, https://www.cellmarque.com). We pretreated the slides with heat-induced epitope retrieval with a citrate-based buffer (Biocare Medical). We ran appropriate negative controls in parallel, using normal rabbit serum in place of the primary antibody. We validated cross reactivity of the anti–SARS-CoV antibody with SARS-CoV-2 by testing controls created from SARS-CoV-2–infected Vero cells embedded with normal human tissues; we used this control as the positive control for subsequent IHC assays. The SARS-CoV nucleocapsid antibody did not cross-react with influenza A(H1N1) virus, influenza B virus, respiratory syncytial virus, parainfluenza virus type 3, human coronavirus (HCoV) 229E, or MERS-CoV in PCR-confirmed tissue samples.

Streptococcus spp. Immunohistochemistry

IHC for the Streptococcus spp. in the tiger utilized Streptococcus antibody raised against *Streptococcus pneumoniae* mouse monoclonal antibody (ThermoFisher, catalog number MA1-83478) which the laboratory reported cross-reactivity with other streptococcal species.[15] Sections were pretreated with proteinase K and utilizes the Mach 4 Universal AP Polymer Kit (Biocare Medica) with Permanent Red Chromogen (Cell Marque/Millipore Sigma).
